# Dissolution Enhancement of Atorvastatin Calcium by Cocrystallization

**DOI:** 10.15171/apb.2019.064

**Published:** 2019-10-24

**Authors:** Reham Al-Kazemi, Yacoub Al-Basarah, Aly Nada

**Affiliations:** Department of Pharmaceutics, Faculty of Pharmacy, Kuwait University, Health Sciences Center, Gabriya, Kuwait.

**Keywords:** Atorvastatin, Glucosamine, Nicotinamide, Cocrystallization, Characterization

## Abstract

***Purpose:*** To enhance the dissolution rate of the poorly soluble drug atorvastatin calcium (ATC) by cocrystallization with selected coformers. Enhancement of the dissolution rate and solubility of the drug, which is classified as Class II of the Biopharmaceutical Classification System (BCS), is expected to enhance the bioavailability.

***Methods:*** Two methods were used for preparing the cocrystals, solvent drop grinding (SDG) and solvent evaporation (SE) method using 1:1, 1:3, and 1:10 drug-coformer molar ratios. Glucosamine hydrochloride (GluN) and nicotinamide (NIC) were investigated as coformers. The cocrystals, their physical mixtures, and the raw ATC were characterized by fourier transform infrared (FTIR spectroscopy), differential scanning calorimetry (DSC), powder X-ray diffraction (PXRD), mass spectroscopy (MS), scanning electron microscopy (SEM), solubility, and dissolution rate studies.

***Results:*** SDG and SE were effective in improving the dissolution rate of ATC with both coformers. Drug: coformer ratio 1:3 was optimum. The solubility values for ATC, GluN-, and NIC-cocrystals were 26, to 35 and 50 µg/mL, respectively. The dissolution rate of ATC from cocrystals was > 90% after 5 minutes, compared to 41% untreated ATC.

***Conclusion:*** Cocrystallization significantly improved the solubility and dissolution, in comparison to the untreated ATC.

## Introduction

The role of pharmaceutical development scientists cover all activities needed to turn an active pharmaceutical ingredient (API) into a marketable product that should be suitable for handling, distribution, and administration to patients. Therefore, in modern pharmaceutical development, solubility and dissolution rate enhancement technologies for poorly water-soluble (PWS) drugs are becoming crucial to improve the bioavailability of drug candidates.^1^

Oral drug delivery still remains the most favorable route of administration, being very convenient for patients, painlessly administered with comparably low costs. It is facilitated by a wide variety of dosage forms.^2^ However, the oral bioavailability is often low and variable, since the process of drug absorption from the GIT depends on many physiological factors such as GI motility, pH, efflux transporters, and pre-systemic metabolism. In addition, food intake, pharmaceutical formulation design and the physicochemical properties of the drug also influence its oral bioavailability.^3,4^ Consequently, understanding the physicochemical properties of a compound is essential to have rational and streamlined formulation process. Furthermore, the aqueous solubility and intestinal permeability are crucial for defining the extent of oral bioavailability.^1^

It has been shown that only 5% of new chemical entities (NCEs) belong to Class I of the Biopharmaceutics Classification System (BCS), having high solubility and high permeability, while 90% of NCEs are poorly soluble in combination with either low or high permeability (BCS II and IV). Since permeability through GI mucosa is high for BCS class II compounds, they are more promising candidates for successful developing and marketing, compared with BCS class IV members.

Different approaches are reported to enhance the solubility of PWS drugs were reported, which generally depend on the properties of the PWS drugs, nature of selected excipients, and nature of intended dosage form. Solubility improvement techniques include physical/ chemical modifications of the drug substance, and other techniques. Physical modification techniques include particle size reduction, e.g. micronization and nanosuspension, solid dispersions, drug dispersion in carriers like eutectic mixtures, solid solutions, and crystal habit modification like polymorphs, amorphous form and cocrystallization. Recently, the latter gained considerable interest and the FDA issued a draft guidance on pharmaceutical cocrystals (PCs) defining a PC to be composed of an active pharmaceutical ingredient and an appropriate coformer. The cocrystals are formed because of several types of interactions including hydrogen bonding, π-staking, or Van der Waals forces.^5^ The formation of cocrystals offers a promising method to modify the physicochemical properties of an API without the need to apply chemical covalent modifications of the API. In addition, the involvement of chemical modification for the API through medicinal chemistry will require extensive and time-consuming toxicological and clinical testing of the resulting new compound(s). Accordingly, PCs offer an opportunity to address challenges of low solubility and other properties of APIs with relatively low cost. Cocrystals may own manufacturing properties and advantages not necessarily related to therapeutic considerations but can lead to a successful commercialization of the API. These properties include improved flow properties, less caking formation, better drying, lower hygroscopicity, and greater processing stability.^6^ Finally, PCs represent an opportunity to patent promising new solid forms of APIs, because a new cocrystal is a new composition of matter between an API and a coformer having a unique binding interaction.^6^

Atorvastatin calcium (ATC) is a model for poorly soluble drug belongs to Class II of the BCS. The drug is a competitive, reversible inhibitor of 3-hydroxy-3-methyl-glutaryl-coenzyme A (HMG-CoA) reductase which catalyzes the conversion of HMG-CoA to mevalonate, an early rate-limiting step in cholesterol biosynthesis.^7^ ATC is rapidly absorbed after oral administration and reaches peak concentration within 1-2 hrs. However, it has poor oral bioavailability (12%), which is a result of low aqueous solubility and high hepatic degradation by first-pass metabolism.^8^ In addition, its bioavailability is highly variable due to instability in acidic media.^9^ Many approaches have been developed to improve the solubility of ATC, including: salt formation, solid dispersion technique using mannitol, PEG-4000, PEG-6000, and PVP-K30,^10^ nanosuspension,^11^ complexation with cyclodextrins,^12^ self-emulsifying drug delivery system,^13,14^ conjugation with chitosan,^15^ and nanosuspension incorporated transdermal patch.^16^

The aim of this study was to prepare cocrystals of the drug with each of these materials: glucosamine (GluN) and nicotinamide (NIC) as coformers in different molar ratios to enhance the dissolution and solubility of ATC. Two methods of cocrystallization were investigated: solvent drop grinding (SDG) and solvent evaporation (SE).

## Materials and Methods

### Chemicals and Reagents

Methanol, acetonitrile, and methanol HPLC grade (Merck, Germany); ATC (atorvastatin calcium trihydrate), GluN (glucosamine HCl) (Zhengzhou Sigma Chemical Co., Ltd., China); nicotinamide (Hubei Ocean Biotech Co., Ltd., China); potassium phosphate monobasic (Loba Chemie, India). All other chemicals were analytical reagent grade.

### Preparation of ATC cocrystals and physical mixtures (PMs)

The SDG and SE methods were used to prepare ATC cocrystals with each of GluN and NIC as coformers.

#### Preparation of ATC cocrystals by SDG method

Predetermined concentrations of the drug and each coformer (GluN and NIC) were mixed and ground for 20 minutes by a mortar and pestle to yield three drug:coformer molar ratios (1:1, 1:3, and 1:10). Few drops (4-20 μL) of 25% methanol/H_2_O mixture per 100 mg of the solid mixture were added to the powder mix while grinding.^17^ The solid powder was then scratched from walls of the mortar and stored in a desiccator containing silica gel as a desiccant for 48 hours to remove traces of the solvent. Subsequently, the powder was passed through a 250 μm sieve to get uniformly size particles and stored in a glass vial inside a desiccator over silica gel till further use.

#### Preparation of ATV cocrystals by SE method

Aliquots from the drug and each coformer, 1:1, 1:3, and 1:10 drug:coformer molar ratios, were dissolved together in 100 mL of 25% methanol/H_2_O mixture, stirred for 5 minutes and left in a Petri dish to evaporate over 3 days at room temperature. The fine crystals obtained were collected into a tight container after being sieved through 250 μm sieve to get uniformly size particles, and stored in a desiccator over silica gel at room temperature till further use.

#### Preparation of ATC coformer physical mixtures (PMs)

In order to evaluate the effect of the cocrystals, physical mixtures in the same drug:coformer molar ratios (1:1, 1:3, and 1:10) were prepared by homogeneously mixing ATC and each of GluN and NIC in a mortar. The PMs were collected, sieved through 250 μm sieve and stored in vials in a desiccator over silica gel at room temperature to prevent adsorption of moisture from the surrounding air till further use. The codes of all cocrystals and their physical mixtures are listed in Table 1.

**Table 1:** Codes of ATC-GluN and ATC- NIC cocrystals in various molar ratios

**Coformer used**	**Method**	**Drug: coformer ratio**	**Code**
GluN	SDG method	1:1	GL1
		1:3	GL2
		1:10	GL3
	SE method	1:1	GS1
		1:3	GS2
		1:10	GS3
	Physical mixture	1:1	GP1
		1:3	GP2
		1:10	GP3
NIC	SDG method	1:1	NL1
		1:3	NL2
		1:10	NL3
	SE method	1:1	NS1
		1:3	NS2
		1:10	NS3
	Physical mixture	1:1	NP1
		1:3	NP2
		1:10	NP3

### Evaluation of the physicochemical properties of the cocrystals and PMs

The prepared cocrystals were evaluated for drug content by UV spectrophotometry to ensure homogeneous distribution of ATC within the powder cocrystals/PMs before the dissolution studies. The dissolution study was performed in triplicate on the prepared cocrystals and their analogous physical mixtures. Further, the cocrystals with the best dissolution results were selected for physicochemical characterization by solubility studies, FT-IR, DSC, and PXRD. All raw materials (ATC, GluN, and NIC and their mixtures/cocrystals) were passed through 250 μm sieve before each experiment.

#### UV analysis of drug content

Calculated amounts from the prepared cocrystals and physical mixtures, equivalent to 10 mg of ATC, were weighed accurately, and dissolved in 10 mL of methanol. Then, 0.1 mL of the solution was diluted with 10 mL phosphate buffer pH 6, filtered and drug content was estimated by UV spectrophotometry (Shimadzu UV-1601, Shimadzu Corp, Japan) at 246 nm for cocrystals with GluN and at 284 nm for cocrystals with NIC. The analysis of the drug content was performed in triplicate for each sample.

#### Solubility studies (Saturation solubility)

The saturation solubility of ATC was determined by adding excess amount equivalent to 35 mg ATC from ATC raw material, cocrystals, and the corresponding physical mixtures, to screw-capped conical flasks containing 40 mL of water. The suspensions were continuously stirred at 200 rpm in an incubated horizontal shaker (Lab Companion, Jeio Tech, Korea) at 37 ± 1°C. The equilibrium solubility was achieved after 72 hours. Afterwards, approximately 0.5 mL from the slurry was withdrawn, and filtered through 0.45 μm membrane filters. The filtrates were appropriately diluted with distilled water, so that the absorbance fell within the range of the standard curve, and analyzed spectrophotometrically as described above for the dissolved drug. An equal volume of water was immediately added into the conical flasks after sampling. The solubility data was expressed as the average of three measurements for each preparation.

#### Dissolution rate study

Dissolution study was performed using USP apparatus II (paddle method). The dissolution test was carried out using 500 mL of phosphate buffer pH 6.^18^ The equivalent of 10 mg ATC was sprinkled into the dissolution flask, employing a stirring speed of 50 rpm and maintaining the temperature at 37± 1°C for 30 minutes. The samples were withdrawn after 5, 10, 15, 20, and 30 min, and the dissolution medium was then replaced after each time interval in order to maintain sink condition. The samples were filtered using 0.45 μm filters and the absorbance was determined using UV spectrophotometer at 246/284 nm. The dissolution study was performed in triplicate.

#### Fourier transform infrared (FT-IR)

Infra-red spectra of ATC, GluN, and NIC were obtained employing Thermo Scientific Nicolet iS50 FT-IR (USA) using attenuated total reflectance (ATR) sampling station. The samples were scanned from 4000 to 400 cm^–1^ at room temperature, using a resolution of 0.5 cm^-1^.

#### Differential scanning calorimetry (DSC)

DSCanalyses of the drug, coformers, and cocrystals were carried out using Netzsch DSC 204 F1 Phoenix (NETZSCH-Gerätebau GmbH, Germany). The heating rate of 10°C/min was employed over a temperature range of 20 – 500°C with nitrogen purging as previously described by Shete et al.^19^ Powder samples (~5 mg) was weighed in aluminum pans with pierced lids, subsequently the endothermic transitions and melting onset were reported.

#### Powder x-ray diffraction (PXRD)

The PXRD analysis of ATC, GluN, NIC, as well as the cocrystals and their respective physical mixtures were undertaken using Bruker D8 ADVANCE X-ray diffractometer (Bruker, Germany) equipped with a Lynxeye detector. The PXRD patterns were collected in angular range between 5 and 70°, with a 2θ scan step of 0.015° and step time of 0.2 second, using CuKα radiation (λ=1.5406Å) at a voltage of 40 kV and a current of 40 mA. The intensity of the reflected radiation is recorded and the data is then analyzed for the reflection angle to calculate the inter-atomic spacing (d value).

#### Mass spectroscopy (MS)

The MS analysis of ATC, GluN, NIC, as well as the cocrystals and their respective physical mixtures were investigated using high-resolution GC-MS DFS (Thermo, USA) under the following conditions: resolution (1000@5%height), electron energy (-70.1 eV), source temperature (175 °C), filament current (3.52 A), scan range (50–900 Da), scan rate (2.0), mass analyzer (Magnetic Sector). The charged particles resulting from the sample were accelerated to the electron multiplier through magnetic field and electric field, then separated according to their mass/charge ratio (m/z). X-Caliber software processed these data to the corresponding mass spectrum.

#### Scanning electron microscopy (SEM)

The surface characteristics of ATC, GluN, NIC, as well as the cocrystals and their respective physical mixtures were studied by SEM (Jeol JCM-5700, Japan). Powder samples were mounted onto stubs using double sided carbon adhesive tape and sputter coated with a thin layer of gold. The specimens were scanned with an electron beam of acceleration potential of 10 kV with a working distance (12-14 mm).

### Methods of analysis and validation

The UV spectrophotometer was used in this study for determination of ATC content in different powder preparations, solubility studies, and dissolution rate studies. The UV method was validated for linearity, precision, accuracy, and sensitivity according to the recommendations of the ICH.^20^

A standard solution of ATC in methanol (20 μg/mL) was scanned in the entire UV range to determine the maximum wavelength of absorbance (λmax) of the drug (246 nm).

Furthermore, series of standard solutions were prepared by taking aliquots of stock solution in methanol (20 μg/mL) and diluted with water/phosphate buffer pH 6 to obtain the concentration in the range of 6-20 μg/mL. The absorbance values of the resulting solutions were measured at 246 and 284 nm for GluN- and NIC- containing cocrystals respectively, since NIC possesses no significant absorbance at 284 nm, thereby avoiding potential interference with the readouts.^21^

The developed UV spectrophotometry methods were validated for linearity (6 to 20 μg/mL), repeatability, precision (intra‐day and inter‐day), accuracy, limit of quantitation and limit of detection.^20,22^

### Statistical analysis

All statistical tests were done using IBM SPSS Statistics 22 software. The results (n=3) were expressed as the mean ± SD. The *t*-test was used to analyze the results of the prepared cocrystals. The value of *P* ˂ 0.05 was considered to be significant. The best results (with significant difference) of the prepared cocrystals with each coformer were selected for further studies.

## Results and Discussion

As there is no new covalent bond formed in ATC cocrystals compared to its mother molecules (ATC and GluN/NIC), the chemical properties of ATC will be well preserved.^6^ However, the crystal structure of ATC co-crystals is totally different from the crystals of the mother compounds. Accordingly, the co-crystal would have physicochemical properties different from the mother crystals. The observed changes in these physicochemical properties will be presented and discussed hereafter.

### Dissolution rate

The dissolution rate studies focus on the initial rate of dissolution, which has been shown to well correlate to the overall drug absorption and bioavailability.^23^ The existence effect of the coformers as simple physical mixtures or cocrystals with the drug on dissolution rate of ATC will be also discussed. Particle size was controlled by sieving all samples through a sieve of 250 μm before using in the dissolution tests.

#### Cocrystals

**Figure 1:**
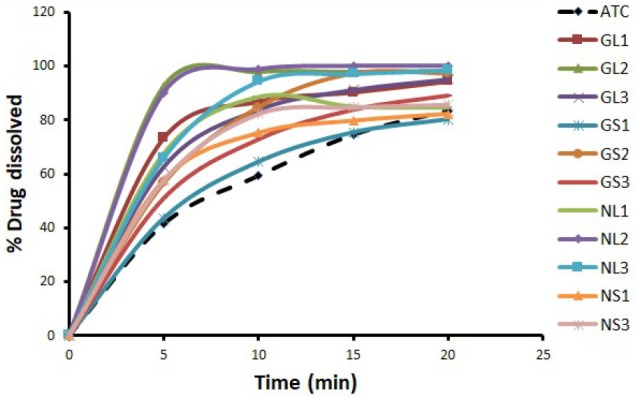
Dissolution rate of ATC with GluN and NIC as coformers using SDG and SE methods in different molar ratios.

The release profiles of ATC from of ATC-GluN and ATC-NIC cocrystals with different ratios applying the two methods of preparation (SDG and SE) are shown in Figure 1.

It is clear from the dissolution profiles that ATC alone showed poor dissolution behavior. When ATC cocrystals were prepared by SDG and SE in different ratios (1:1, 1:3, 1:10) with GluN, an improvement in the dissolution rate was observed (Figure 1). Using SPSS software, GL2 cocrystal was the only preparation that had a significant difference (*P* < 0.05) when compared to the untreated ATC dissolution results. Approximately 98% of ATC released after only 10 min in GL2 preparation, compared to <60% in case of untreated ATC. This indicates that co-grinding of ATC with GluN while adding small amount of the solvent can significantly improve dissolution efficiency of ATC. Figure 1 also points out that SDG method (GL1, GL2, and GL3) with different ratios using GluN as a coformer resulted in dissolution rates higher than the SE method (GS1 and GS3) and a similar dissolution rate to GS2. It could be concluded from the above results that better dissolution rates were observed for samples containing higher ratios of GluN. However, no further increase in the dissolution rate was found when the ratio of GluN was increased from 1:3 to 1:10 molar ratio of drug:coformer, indicating that there is an optimum ratio for GluN to increase dissolution rate of ATC. This is consistent with the data reported by other researchers, where the dissolution properties of carbamazepine (CBZ) was improved by co-grinding CBZ with GluN, but there was an optimal ratio of the carrier. Above this ratio, the authors observed a decrease in the dissolution rate of CBZ. This was explained by increased viscosity around the CBZ particles due to dissolving large amounts of the hydrophilic carrier (GluN).^24^

Figure 1 shows that all NIC preparations enhanced the dissolution rate of ATC, but higher notably in case of the cocrystal prepared by SDG in 1:3 molar ratio. NL2 was the only preparation that exhibited significant difference (*P* ˂ 0.05) when comparing its dissolution rate with the untreated ATC. More than 98% of ATC was released after 10 min from NL2 preparation, while less than 60% was released from the untreated ATC. This indicates that SDG method using NIC as a coformer in 1:3 molar ratio can significantly improve the dissolution rate of ATC. In general, using SDG method with NIC as a coformer in NL2 and NL3 preparations showed better dissolution results when compared to SE, as in NS1, NS2, and NS3. NL1 cocrystals exhibited very close dissolution results to NS2. Furthermore, application of low molar ratio of NIC to ATC (NL1 and NS1) caused a reduction in the dissolution rate when compared to higher ratios of the coformer (NL2 & NL3) (Figure 1). Although higher dissolution rates were achieved by increasing the ratio of NIC in the two methods, no further increase in the dissolution rate was observed when the molar ratio of NIC was increased from 1:3 to 1:10, drug:coformer. These results are in agreement with dissolution profiles of artemisinin-NIC solid dispersions that were prepared at various drug-carrier ratios, whereby increasing NIC ratios enhanced the dissolution rate up to certain limit, followed by decrease in dissolution rate with higher ratios.^25^

**Figure 2:**
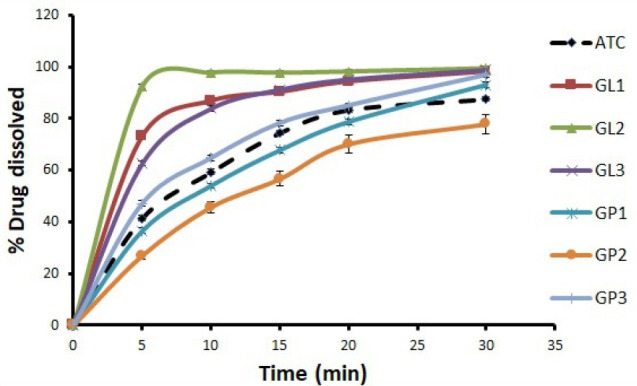
Dissolution rate of ATC cocrystals with GluN as a coformer using SDG method in different molar ratios, compared to the PMs..

**Figure 3:**
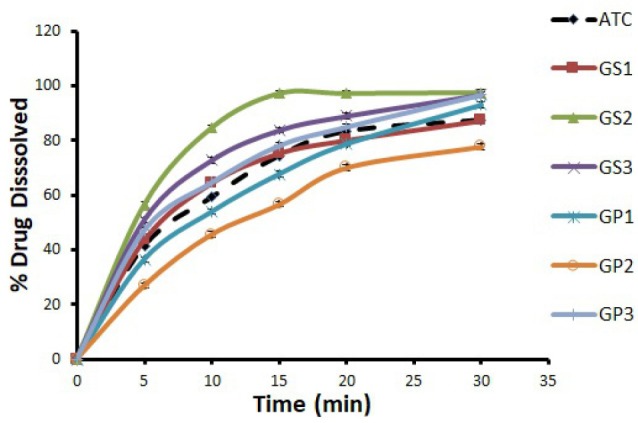
Dissolution rate of ATC cocrystals with GluN as a coformer using SE method in different molar ratios, compared to the PMs.

The dissolution rate profiles of ATC from preparations of ATC-GluN and ATC-NIC using SDG and SE methods along with their physical mixtures were investigated. The effect of the 2 coformers were similar, and therefore the results were exemplified by ATC-GluN using SDG and SE, in Figures 2 and 3, respectively. The data demonstrate that all prepared cocrystals by the two methods were better than their physical mixtures in improving the dissolution rate of ATC. In both SDG and SE methods, 1:3 molar ratio was the best among other ratios. Furthermore, a non-significant increase (*P* > 0.05) in the dissolution rate was observed when cocrystals were prepared using SDG method, compared to SE method. During grinding of GluN or NIC with ATC in SDG method, coformers’ particles were enforced mechanically between ATC particles and produced better dissolution efficiency. Therefore, in the current study SDG is recommended as the method of choice to enhance the dissolution rate of ATC. It is worthy to mention that the two methods can improve the dissolution rate of ATC, however relatively higher cost may be associated when using SE method, where larger amount of solvent is used. In many research studies, SDG method was found to be highly efficient for the synthesis of cocrystals, resulting in fast and quantitative formation of cocrystals.^17^

#### Physical mixtures

With regard to the dissolution rate of ATC from the PMs, Figures 2 and 3 showed that the presence of GluN had no significant positive effects on the dissolution rate of ATC as with GP3, or even less than the dissolution rate of untreated ATC as with GP1, and GP2. Likewise, similar patterns were obtained for NIC (data not shown). The presence of GluN and NIC as physical mixtures with ATC may reduce the accessibility of water to ATC, which magnified the local effect of GluN and NIC on the diffusion layer leading to reduce dissolution rate of ATC.^26^ In addition, the physical mixtures exhibited slower dissolution rates, compared to their corresponding cocrystals, because of the weak interparticular bonding with the coformer in physical mixtures and the higher degree of crystallinity, compared to the corresponding cocrystals. The preferential attraction of water molecules by the hydrophilic coformers would also deprive the ATC molecules to be engaged in H-bonding with water and thus remain undissolved. Therefore, the presence of coformer as a physical mixture with the drug (ATC) did not enhance its dissolution rate in comparison with the cocrystal preparations.

Karki and coworkers investigated the formation of NIC (as API) cocrystals with dicarboxylic acids (as coformer) using solution crystallization, crystallization from the melt, and liquid-assisted grinding methods. The authors found that mechanochemical methods were more efficient in screening for stoichiometric variations of cocrystals. In addition to efficiency and speed, SDG provided also a highly crystalline product.^27^ In many previous studies, the dissolution rate enhancing effect of NIC was accredited to its hydrotropic effects, where it is hypothesized to interact with solvent (water) to increase the ATC solubility.^28^ However, our study indicated that in the physical mixture samples, NIC had no positive effect on the early dissolution rate of ATC. The same behavior was also seen with the physical mixtures of GluN. These findings substantiated the assumption that these coformers were not acting like hydrotropes and the enhancement in dissolution results may be due to the impact of complexation and hydrogen bond formation between the drug and the coformer.

#### Effect of coformer type on dissolution enhancement of ATC

GluN and NIC were used as coformers to enhance the dissolution rate of ATC and both improved the dissolution rate significantly, compared to the untreated ATC. In general, the increase in dissolution rate was more evident in case of NIC compared to GluN, however, NL2 exhibited non-significant higher dissolution rate than GL2 (Figure 1). The magnitude at which coformers improve the dissolution rate was dependent on type of coformer, coformer ratio, and physicochemical properties of the coformer and the drug. In addition, it depends on the magnitude of total energy drop due to inclusion of the hydrophobic drug in NIC and GluN aggregate. This change in total energy of the system was related to hydrogen bonds and interactions between the coformers and hydrophobic drug.^29^

### Saturation solubility of ATC

**Figure 4:**
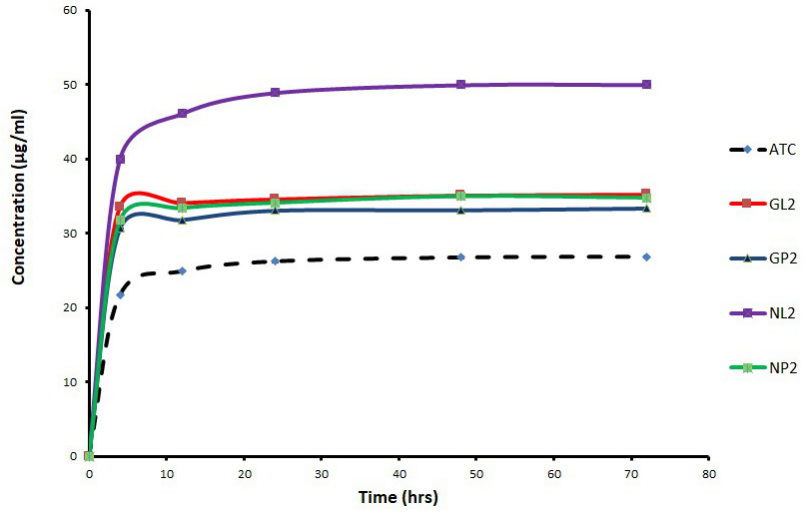
Solubility of ATC from the different preparations

The saturation solubility of the selected cocrystals and their physical mixtures was studied in water (Figure 4). The solubility of ATC was improved significantly (*P* ˂ 0.05) from the cocrystal preparations GL2 and NL2, as well as from their physical mixtures. The ATC solubility from NL2 was the best among all preparations including the physical mixtures and the untreated ATC. NIC is well known as hydrotropic agent, and its ability to solubilize wide variety of therapeutic compounds has been demonstrated previously.^25^ The results of saturation solubility indicated that the saturation solubility of the cocrystals was enhanced by 31.05% (GL2) and 86.19% (NL2), compared to the untreated ATC. In GP2 and NP2 physical mixtures, an enhancement in saturation solubility was also observed and amounted to 24.16 % and 29.49 % respectively.

The improvement in saturation solubility can lead to dissolution rate enhancement, nevertheless the dissolution rate remains the most important factor affecting the drug bioavailability.^30^ In case of the physical mixtures, the dissolution rate data proved that an improvement in the saturation solubility cannot be correlated directly to early dissolution rate enhancement. Therefore, it could be concluded that other parameters must be responsible for the enhanced dissolution rate of the cocrystals. Several theories have been proposed to show how hydrotropes like NIC and GluN enhance the solubility of poorly soluble drugs. Among other mechanisms, formation of complexes is widely accepted mechanism in the literature.^29^ The wettability effect of the hydrophilic coformers can positively affect the solubility of ATC in cocrystals and physical mixtures but not the early dissolution rate of ATC, as shown in our study with the physical mixtures. In addition, the subsequent DSC, PXRD, FT-IR, and MS studies showed changes in ATC physicochemical properties within the cocrystals, which was a proof of molecular interaction of the coformers with ATC. This may also explain the observed increased solubility and dissolution of the prepared cocrystals.

### Solid state characterization

#### FT-IR spectroscopy

**Figure 5:**
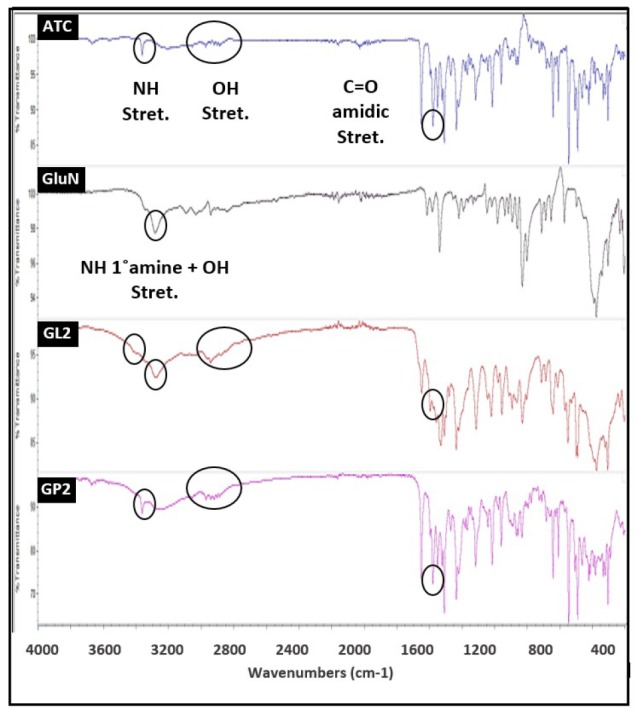
FT-IR spectra of pure ATC, glucosamine (GluN), GL2 cocrystals (1:3, drug: GluN) prepared by SDG method, and GP2 physical mixture (1:3, drug: GluN).

FT-IR spectroscopy has been used to investigate drug and coformer interactions, which may lead to peak broadening and the bathochromic shift of the absorption bands of interacting functional groups.^31^ FT-IR spectra for ATC, GluN, GL2, and GP2 samples are shown in Figure 5. FT-IR spectrum for ATC showed strong N-H stretch and C=O stretch of amide group at 3368.85 and 1578.01 cm^-1^ respectively. In addition, ATC showed O-H stretching at 3212.23 cm^-1^ and 3055.18 cm^-1^. GluN spectrum exhibited peaks at 3282.69 cm^-1^, corresponding to N-H stretching (1^o^ amine) and O-H stretching. It was apparent from the FT-IR spectra of GL2 sample that this sample was associated with changes at the molecular level, reflected in a broader peak in the spectrum following the intermolecular hydrogen bonding. The transmission peak of ATC at 3368.85 cm^-1^ of N-H stretching was lost and C=O stretch of amidic group of ATC was shifted from 1578.01 cm^-1^ to 1595.33 cm^-1^ with a change of the peak shape. Also, the spectrum of GL2 exhibited a significant change of C-OH stretching alcoholic group at 3212.23 cm^-1^ and 3055.18 cm^-1^. These changes were related to weakening or removal of these stretching frequencies, which were strong evidence of H-bonding between the drug and the coformer (GluN). The hydrogen bond donors and acceptors groups in ATC molecule can interact with the primary amine and the unsubstituted sugar hydroxyl groups of GluN forming H-bonds. Similarly, Chadha and other coworkers found that in FT-IR spectrum of efavirenz, cocrystals with oxalic acid, the N-H and C=O stretch of efavirenz and the C=O stretch of oxalic acid were shifted. This finding suggests that both N-H and C=O of amidic group of efavirenz and C=O of carboxylic acid group of oxalic acid are participating in hydrogen bonding in efavirenz-oxalic acid cocrystals.^32^ On the other hand, the spectrum of the physical mixture (GP2) demonstrated almost every characteristic peak of ATC and the coformer (GluN). The results of physical mixture revealed no considerable changes in the IR peaks of ATC in the physical mixture compared to the untreated ATC, thereby indicating the absence of any interaction, which in turn explained the poor dissolution rate observed with the physical mixtures.

**Figure 6:**
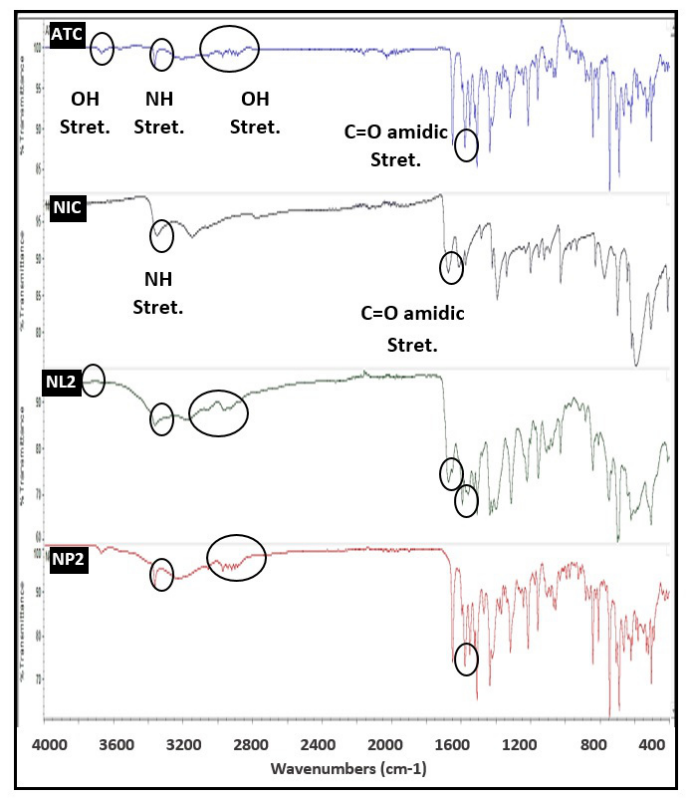
FT-IR spectra of pure ATC, nicotinamide (NIC), NL2 cocrystals (1:3, drug: NIC) prepared by SDG method, and NP2 physical mixture (1:3, drug: NIC)..

FT-IR spectra for ATC, NIC, NL2, and NP2 samples are shown in Figure 6. NIC spectrum exhibited peaks at 3350.21 cm^-1^ (N-H stretching amide), and 1674.2 cm^-1^ (C=O stretching amide). The spectrum of NL2 exhibited a significant difference in the observed vibrational transitions related to weakening or removal of some stretching frequencies. In NL2 spectra, vibrational peak at 3670.52 cm^-1^ (O-H stretching of ATC) disappeared and C=O stretch of amidic group of ATC was shifted from 1578.01 cm^-1^ to 1593.37 cm^-1^ with a change of peak shape, which indicated molecular interaction of ATC with the coformer (NIC). In addition, the three bands of ATC, namely O-H stretching bands at 3212.23 cm^-1^, 3055.18 cm^-1^, and 2971.48 cm^-1^ were shifted to 3177.34, 3057.82, and 2962.36 cm^-1^ with a change of peaks shape. Furthermore, the characteristic band of NIC at 3350.21 cm^-1^ (N-H stretching amide NIC) disappeared, and the peak at 1674.2 cm^-1^ (C=O stretching amide NIC) showed reduced intensity in NL2, which further supports the molecular interaction between the drug and the coformer (NIC). However, disappearance of the peak at 3350.21 cm^-1^ may be due to overlapping of the drug molecules by the presence of the coformer (NIC). The changes in NL2 spectrum are a strong evidence of H-bonding between the drug and the coformer (NIC). Similarly, Setyawan et al came to the same conclusion in the characterization of artesunate-NIC cocrystals, where the loss of C=O stretching, C-H stretching, and O-H bending of artesunate, in addition to loss of secondary amide banding of NIC suggested the presence of hydrogen bonding between artesunate and NIC due to the formation of cocrystals.^33^ On the other hand, the physical mixture (NP2) showed all ATC’s characteristic peak with no considerable changes. This indicates that no significant interaction occurred in the physical mixture of the drug and NIC, which again explains the slow dissolution rate of ATC from the physical mixtures.

#### DSC analysis

DSC analysis is a very useful thermoanalytical method for the characterization of solid-state interactions between drugs and coformers through the appearance, shift or disappearance of endothermal or exothermal effects and/or variations in the relevant enthalpy.^32^ In our study, the thermograms of the cocrystals were different in pattern and intensity, as compared to untreated ATC and the coformers, which indicated their interaction by forming different crystal lattice in the cocrystals (Data not shown, Supplementary Supplementary Figures S1-S4).

DSC analysis of GL2 and GP2 was carried out to study the interactions between ATC and GluN in the solid state. The DSC curve of the physical mixture of atorvastatin with glucosamine (GP2) showed two peaks at 156.6°C and 212.2 °C (Figure S1), corresponding to the melting transitions of the drug and conformer respectively, with shifting of the drug peak to a lower melting transition; where the melting point of ATC was 167.8°C. This indicates a distorted crystallinity of ATC in its physical mixture with GluN. Balata et al came to the same conclusions with the physical mixture of simvastatin and poloxamer 188, where the same endotherms corresponding to both the drug and the carrier melting transitions were observed with shift of the drug peak to a lower melting transition and reduced intensity.^34^ The DSC thermogram for GL2 (Figure S2) showed sharp endothermic peak at 170.2°C suggesting that the drug was still in the stable crystalline form. Moreover, this melting point was different from that of starting components as well as from those appeared in DSC scan of the physical mixture. This shift in the melting point is explained by the change in crystal lattice of ATC in presence of coformer, suggesting the formation of a new phase with relatively different crystal lattice in the cocrystal. This result is consistent with the data reported by Shah et al, where the thermogram of the ritonavir-NIC cocrystals was different in pattern and intensity as compared to ritonavir and NIC which indicates their interaction.^35^

The DSC curve of the physical mixture of atorvastatin with NIC (NP2) showed the melting transitions at 111.3°C and 187°C (Figure S3), which are corresponding to the melting point of ATC and the coformer. The shifts in the melting temperature of NP2 occurred as a consequence of interaction induced by thermal energy between the drug and the conformer, during the DSC scan of the sample.^32^ Alternatively, these may be attributed to change in powder geometry of samples during preparation.^36^ The DSC thermogram for NL2 showed a single sharp endothermic peak at 191.2°C, which was higher than those observed for ATC (167.8°C), NIC (132.5°C), and the physical mixture (NP2) (Figure S4). The difference in the melting point and the appearance of the single peak in NL2 DSC curve confirmed the formation of a new solid phase.^35^ The observed higher melting point might be due the crystal packing nature in ATC-NIC co-crystal, which indicated higher thermal stability compared to the individual components.^37^

The DSC results showed that the melting point of the cocrystals deviated significantly with respect to the melting point of pure drug and the individual coformers suggesting their interaction. This indicates that the melting point of the API may be changed by cocrystallization.^37^ Our study also showed that the melting point of the cocrystals could be highly dependent on the cocrystal formers, since different melting points were found for the two coformers. The melting point of GL2 cocrystals (170.2 °C) was between the melting point of ATC (167.8 °C) and GluN (212.2°C), whereas the melting point of NL2 cocrystals (191.2ºC) was higher than the melting point of ATC and NIC (Table 2). In relation to our findings, Perlovich (2015) found that after analyzing 727 cocrystals, the melting points of 55.3% of the cocrystals were in the range between, 15.8% higher than, and 28.9% lower than those of the individual components.^37^

**Table 2:** Heat of fusion (ΔH) and the peak melting temperature of ATC, GluN, NIC, freshly prepared cocrystals and their physical mixtures

**Sample**	**Peak temperature (˚C)**	**ΔH (J/g)**
ATC	167.8	100.02
GluN	212.9	544.73
NIC	132.5	215.31
GL2	170.2	72.92
GP2	156.6 164.2L 212.2	10.89 3.10/170.24
NL2	191.2	16.77
NP2	111.3 187	103.46 23.27

The heat of fusion (∆H) of these cocrystals and their physical mixtures were calculated and summarized in Table 2. All preparations exhibited lower ∆H values (area under the melting transition) than the pure drug (ATC) and the coformers. The lower ∆H values points out to the increased interactions and miscibility between ATC and the coformers.^38^ The enthalpy change (∆H) was reduced in the cocrystal preparations (GL2 and NL2) and was minimum in NL2 preparation. The maximum decrease in (∆H) was observed with NL2 followed by GL2, NP2, and GP2. These results were in accordance with their dissolution rate, where NL2 resulted in the highest rate of dissolution.

#### PXRD analysis

PXRD was used to measure the degree of crystallinity of ATC cocrystals and the physical mixtures and also to provide information on the solid systems in terms of interaction between materials. Such interactions may produce new diffraction peaks as compared to the constituent materials.^33^ A different PXRD pattern for the cocrystals from those of the individual components confirms the formation of new phase.^32^ In addition, the decrease in crystallinity of API can be a reason for the enhanced dissolution of prepared samples.^39^ Less crystalline compounds have less uniform molecule arrangements than crystalline compounds, and this disorder was the driving force for its greater solubility and faster dissolution rates.^40^ Our results showed that the crystallinity of the cocrystals and the corresponding physical mixtures were decreased obviously when they were compared to the crystallinity of untreated ATC. Although the crystallinity of the physical mixtures decreased, their dissolution rates did not increase in parallel (Figures 2 and 3). This pointed out that the enhancement of dissolution rate of our cocrystals may be due to other reasons rather than only decrease in crystallinity of the cocrystals. In relation to our findings, Balata et al found that all the tested physical mixtures exhibited the same characteristic peaks of both the drug (simvastatin) and the hydrophilic carrier (poloxamer 188) with reduced intensity. This observed reduction in drug crystallinity in physical mixtures did not enhance the dissolution rate of the drug in the physical mixtures, where all the physical mixtures had similar dissolution rate as the untreated drug or less.^34^

**Figure 7:**
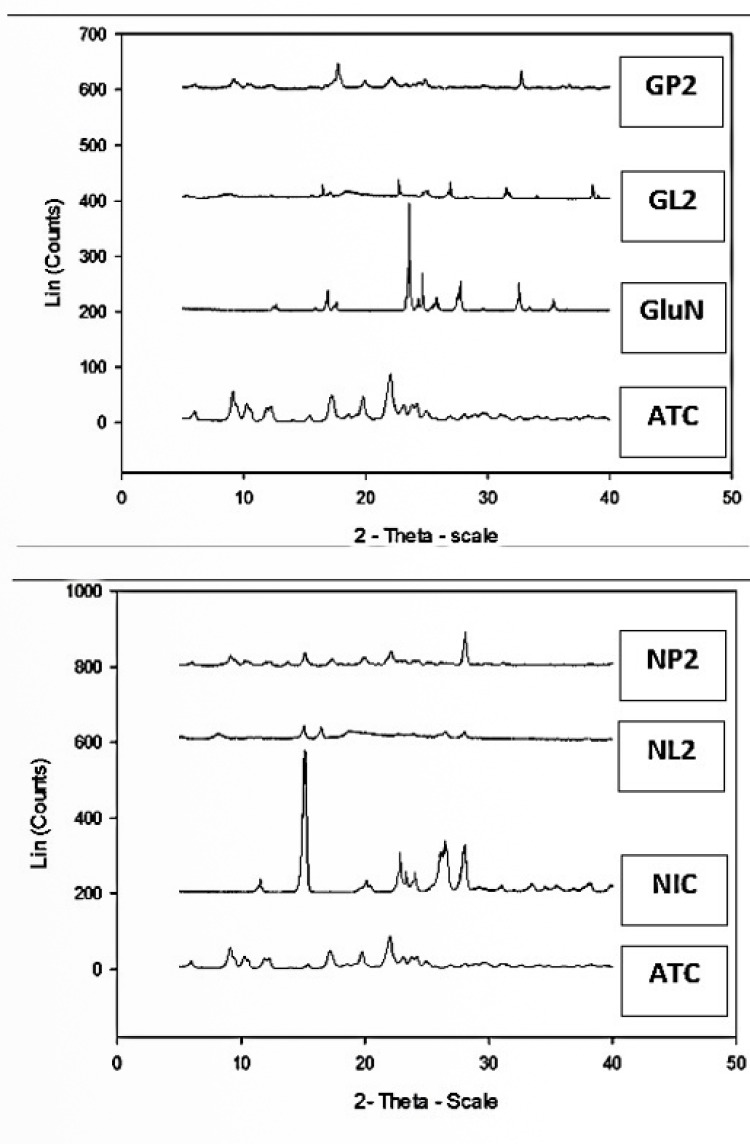
PXRD pattern of pure ATC, atorvastatin cocrystals with different coformers and their physical mixtures.

The diffraction patterns of ATC, GluN, and NIC showed characteristic high-intensity peaks, which indicated that the drug and the two coformers present in crystalline form, and agrees with the DSC results. The results of PXRD analyses for the prepared products GL2 and NL2 and their physical mixtures are shown in Figure 7. In GL2, one new peak appeared at 38.635° (intensity = 97.2%), which was absent in both ATC and glucosamine. The PXRD pattern of NL2 also showed appearance of a new peak at 16.046° (intensity = 98.7%). The PXRD patterns of GL2 and NL2 showed clearly broader peaks with lower intensities. The PXRD patterns of the cocrystals GL2 and NL2 pointed out some characteristic peaks of the coformers, while some characteristic peaks of ATC were significantly lower in intensity and some others disappeared. In addition, the cocrystals revealed diffractograms which were different from the respective physical mixtures as they exhibited more displaced angles as well as different peak intensities, compared to raw ATC and the physical mixtures. This observation is indicative of stronger interaction between the coformers and ATC through cocrystallization.^25^ The formation of new crystalline structure could be confirmed by the significant changes in the diffractograms of cocrystals GL2 and NL2 and the transformation of the crystalline lattices of ATC and coformers. These results stand in accord with our previous DSC results, confirming that a new phase was formed in the cocrystals.

The PXRD pattern of the physical mixtures (GP2) and (NP2) showed the appearance of broader peaks with lower intensities, compared to the peaks of the pure drug and the coformers, nevertheless without any new peak appearance. The PXRD patterns of the physical mixtures (GP2 and NP2) showed the characteristic peaks of the coformers and ATC with significantly lower intensities indicating coformer influence. When comparing PXRD patterns of the prepared cocrystals GL2 and NL2 with their physical mixtures (GP2 and NP2), the physical mixtures showed slightly higher degrees of crystallinity and intensity of peaks vs. the cocrystals.

#### Mass spectroscopic (MS) analysis

Mass spectroscopic analysis was performed for the pure drug (ATC), and the coformers GluN and NIC (Figures S5-7). The molecular ion mass at m/z 306.2 corresponds to ATC. The molecular ion mass at m/z 214.1 corresponds to GluN (for which the molecular mass is 215.63) and that at m/z 122 indicates NIC (for which the molecular mass is 122.12).

Theoretically, complex formation of 1:1 stoichiometric ratio between the drug (ATC) and the coformer (GluN) will result in a product of molecular ion mass of m/z ~520.3. In case of the other coformer (NIC), a complex of molecular mass of ~428.3 was produced. In case of GL2 cocrystals, the presence of MS signal at m/z 523.1 indicated the formation of the molecular complex between atorvastatin and GluN (Figure S8). Also, the signal at m/z 430.3 (Figure S9) indicated the formation of molecular complex between the drug and NIC in NL2 cocrystals. The appearance of separate signals at m/z 306.3 and 214.1 in GL2 mass spectra and separate signals at m/z 306.3 and 122.1 in NL2 mass spectra ensures that the bonding between the drug (atorvastatin) and the coformers (GluN and NIC) was weak hydrogen bonding and consequently dissociated into their constituting molecules during the ionization process. MS analysis of the physical mixtures GP2 (Figure S10) and NP2 (Figure S11) showed no signals corresponding to the complex formation between the drug and the coformers. The presence of mass signal at m/z 306.2 in the physical mixtures GP2 and NP2 proved that the drug remained free in the mixtures without any interaction with the coformers.

#### Scanning electron microscopy (SEM)

**Figure 8:**
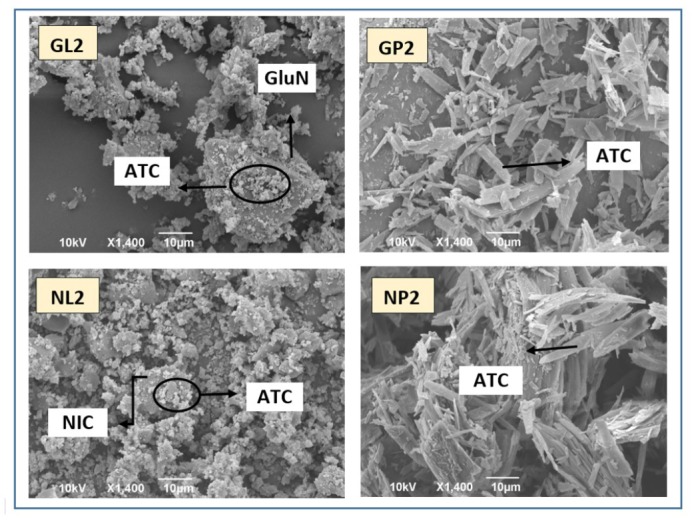
SEM photographs of atorvastatin cocrystals with different coformers and their physical mixtures..

The surface characteristics of ATC, coformers (GluN and NIC), prepared cocrystals (GL2 and NL2), and the corresponding physical mixtures (GP2 and NP2) were studied by SEM (Figure 8). The cocrystals GL2 and NL2 exhibited a significant change in particle shape and surface topography. They were almost irregular spherical in shape and different from those of the pure drug and coformers. The drug particles inside the cocrystal were transformed to smaller crystalline structures, which were finely dispersed and attached to the surface of the coformers particles. These changes in cocrystals shape, especially the spherical and rounded shape, might enhance the flow properties, which was advantageous in formulation as solid dosage forms. In addition, the spherical shape is usually accompanied by increase in surface area and thus leading to improved dissolution rate of these cocrystals as well. Furthermore, the presence of the polar coformers around ATC particles is expected to improve the wettability and dispersion of the originally clumped ATC particles. On the other hand, in the corresponding physical mixtures (GP2 and NP2), ATC particles can be identified as crystalline with irregular rectangular particles, resulting in lower rate of dissolution, which might be correlated to the corresponding cocrystals.

## Conclusion

SDG and SE can be regarded as effective methods in improving the dissolution rate of ATC using GluN and NIC as coformers. SDG method significantly improved the dissolution of ATC, with an optimum 1:3 drug to coformer molar ratio. The prepared cocrystals GL2 and NL2 showed marked increase in the dissolution rate of ATC amounting to 100%, in comparison with 40% for the untreated drug after 5 minutes. The saturation solubility of the cocrystals was enhanced by 31.05 % in GL2 and 86.19 % in NL2, compared to the untreated ATC.

The DSC data indicated change in the crystal properties, which may explain the improvement of ATC dissolution rate. The FT-IR data of the physical mixture (NP2) confirmed the presence of the characteristic drug peaks, while the cocrystals showed a significant difference, compared to the pure drug spectrum. The PXRD patterns of the prepared cocrystals (GL2 and NL2) revealed some characteristic peaks of the coformers, while the characteristic peaks of ATC were of significantly lower intensity or disappeared completely. The appearance of separate MS signals at m/z 306.3 and 214.1 in GL2 mass spectra and separate signals at m/z 306.3 and 122.1 in NL2 mass spectra ensures that the bonding between the drug and the coformers (glucosamine and NIC) was weak hydrogen bonding and they were dissociated into their constituting molecules during the ionization process. MS analysis of the physical mixtures GP2 and NP2 showed no signals indicative of complex formation between the drug and the coformers. The presence of mass signal at m/z 306.2 in the physical mixtures proved that the drug was present free in the mixtures without any interaction with the coformer. SEM showed that the drug particles in the cocrystals were transformed to smaller and rounded crystalline structures, which were finely dispersed and attached to the surface of the coformers particles. In contrast, for the corresponding physical mixtures (GP2 and NP2), the drug particles were very similar to the pure ATC particles which appeared crystalline with irregular rectangular particles.

In brief, the results of this study confirmed the successful approach of formulation of cocrystals as a potential strategy for improvement of the bioavailability of ATC and similar drugs. Furthermore, in vivo assessment of the developed ATC cocrystals is foreseen to confirm this concept.

## Conflict of Interest

Authors declare no conflict of interest in this study.

## Acknowledgments

The authors are highly indebted to the support staff of Pharmaceutics Department, for their indispensable technical support during conducting the Master thesis work of Reham Al-Kazemi. Special thanks to the Science Core Facilities, Faculty of Science, Kuwait University, Kuwait for running the thermal and XRD analyses.

## Supplementary files


Supplementary file 1 contains Figures S1-S11.
