# Supplementary material for: Dissolution Enhancement of Atorvastatin Calcium by Cocrystallization
Source: Adv Pharm Bull. 2019 Oct 24;9(4):559–70. doi: 10.15171/apb.2019.064 (PMC6912187; doi:10.15171/apb.2019.064)
Supplement: Supplementary file 1 — contains Figures S1-S11. [file apb-9-559-s001.pdf]

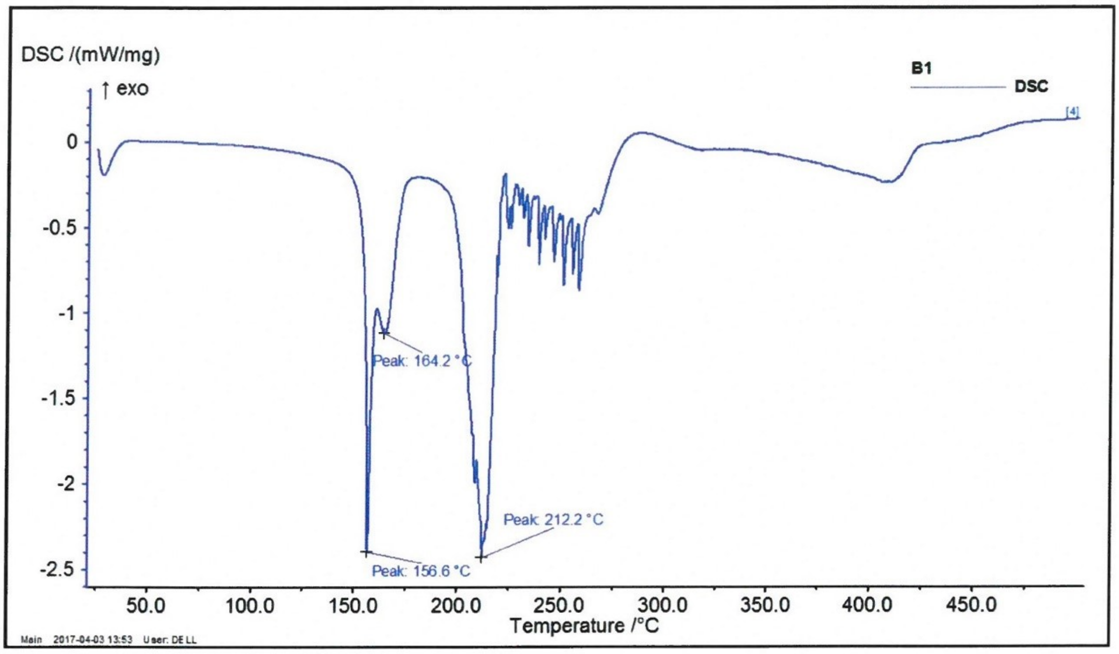

Figure S1. DSC thermogram of freshly prepared GP2 physical mixture (1:3, drug: GluN).

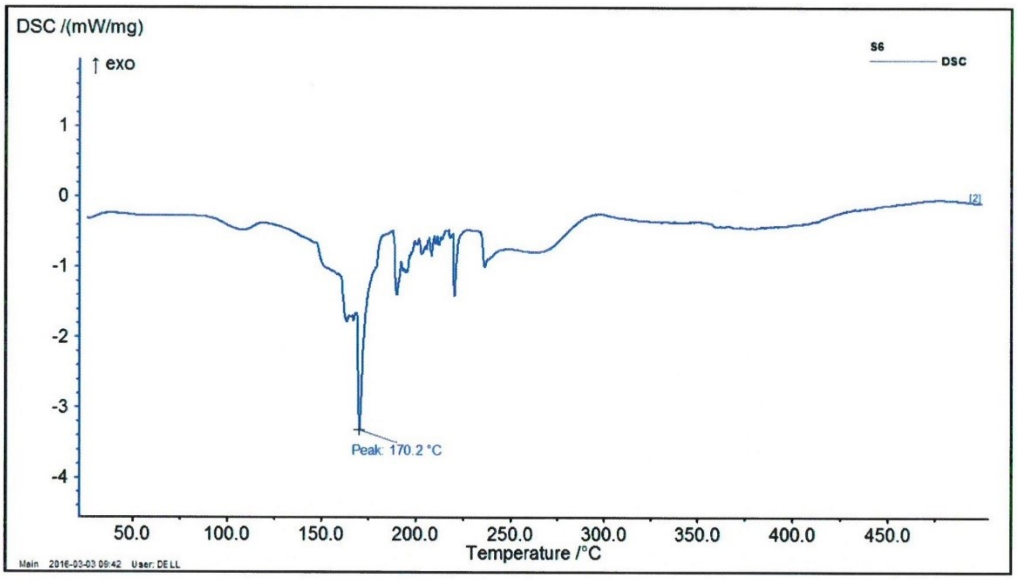

Figure S2. DSC thermogram of freshly prepared GL2 cocrystals (1:3, drug: GluN) prepared by SDG method.

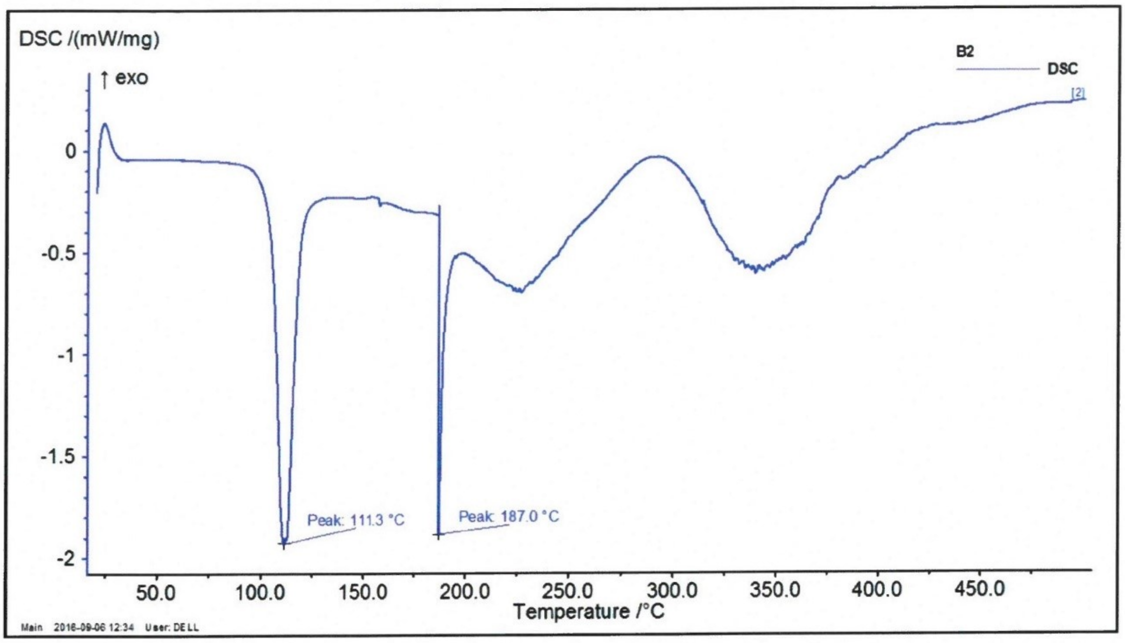

Figure S3. DSC thermogram of freshly prepared NP2 physical mixture (1:3, drug: NIC).

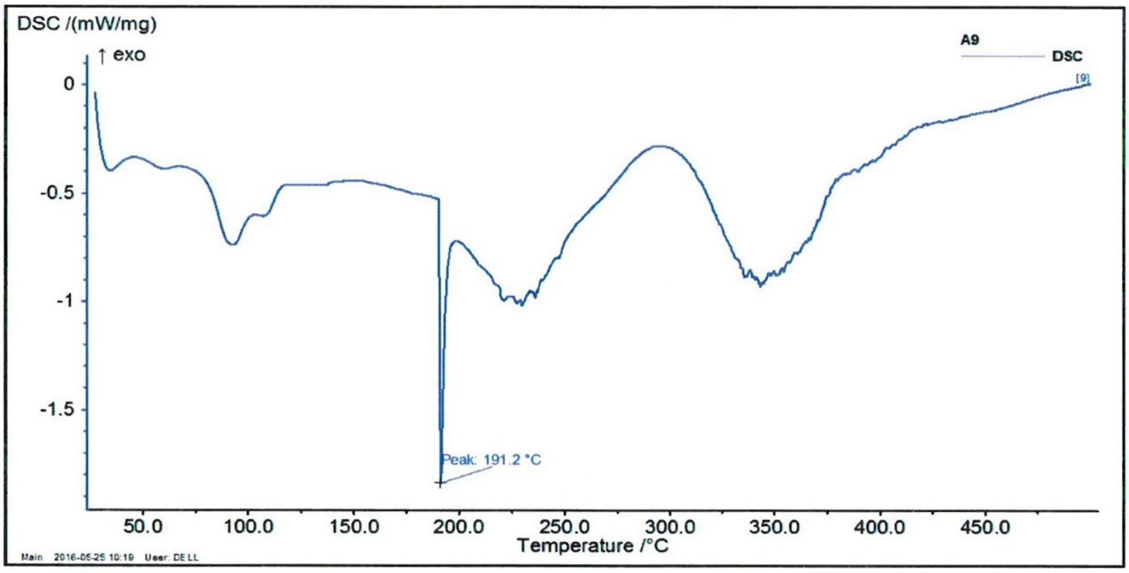

Figure S4. DSC thermogram of freshly prepared NL2 cocrystals (1:3, drug: NIC) prepared by SDG method.

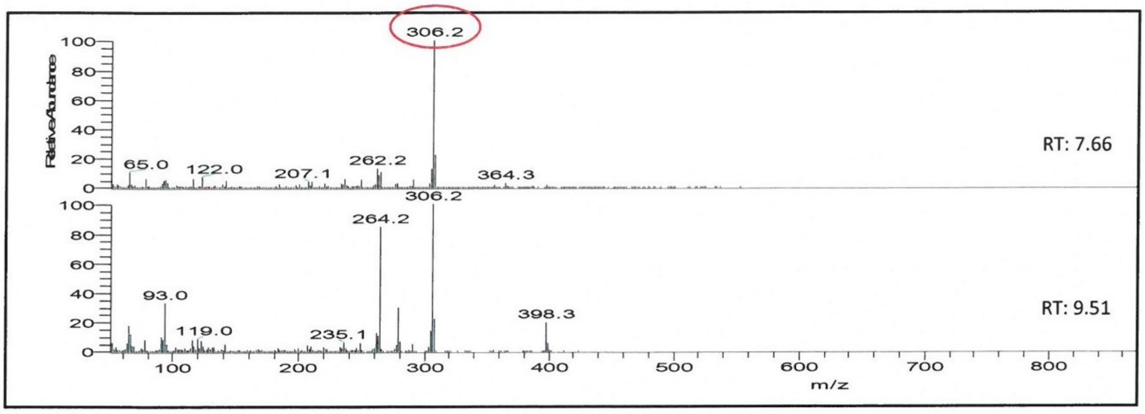

Figure S5. Full mass spectrum of pure ATC.

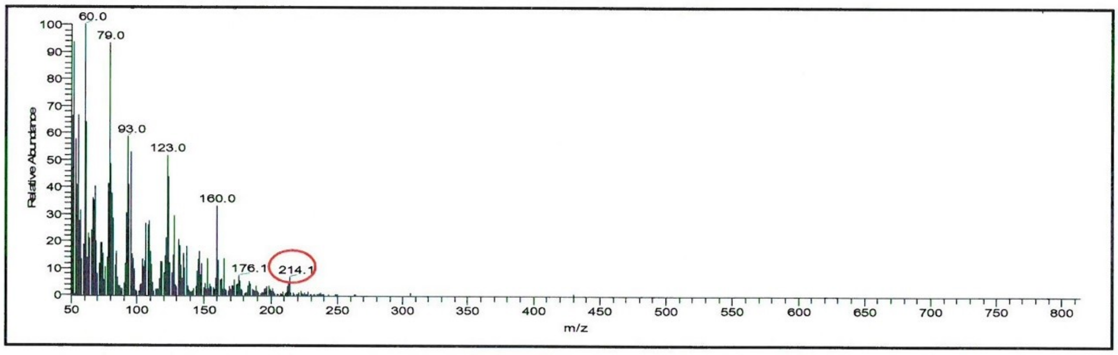

Figure S6. Full mass spectrum of GLU-N.

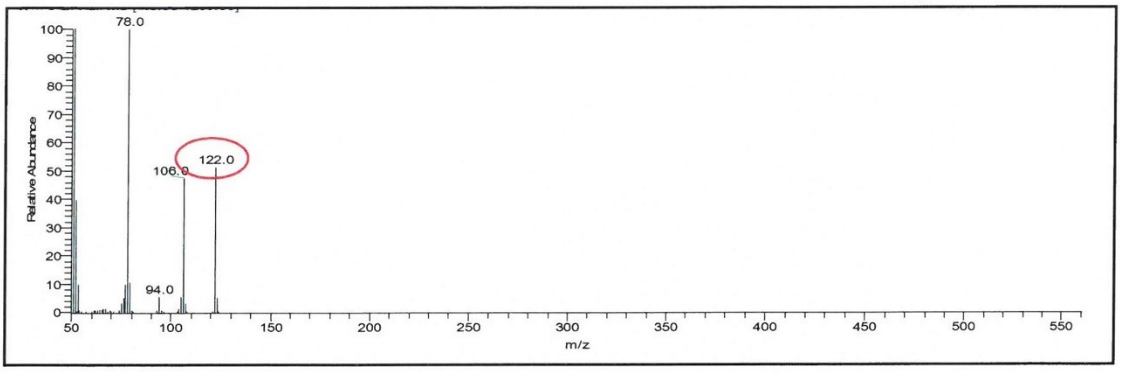

Figure S7. Full mass spectrum of NIC.

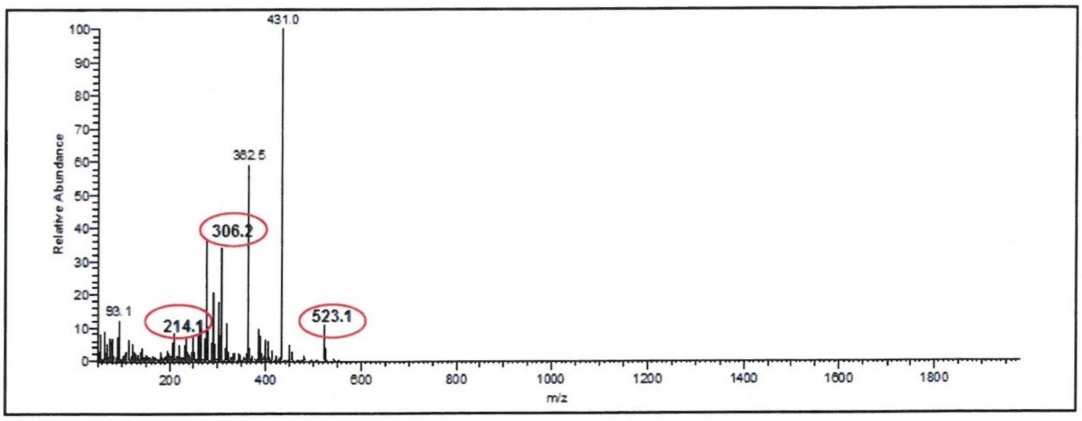

Figure S8. MS analysis of freshly prepared GL2.

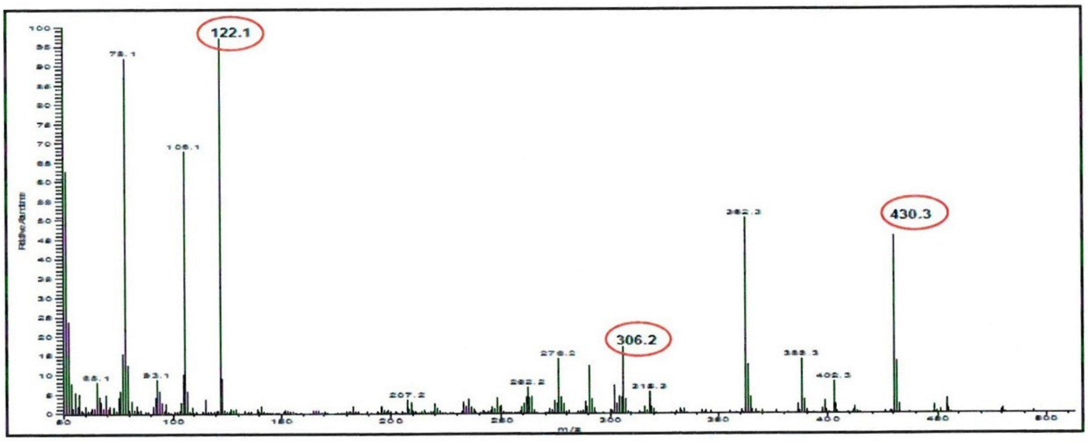

Figure S9. MS analysis of freshly prepared NL2.

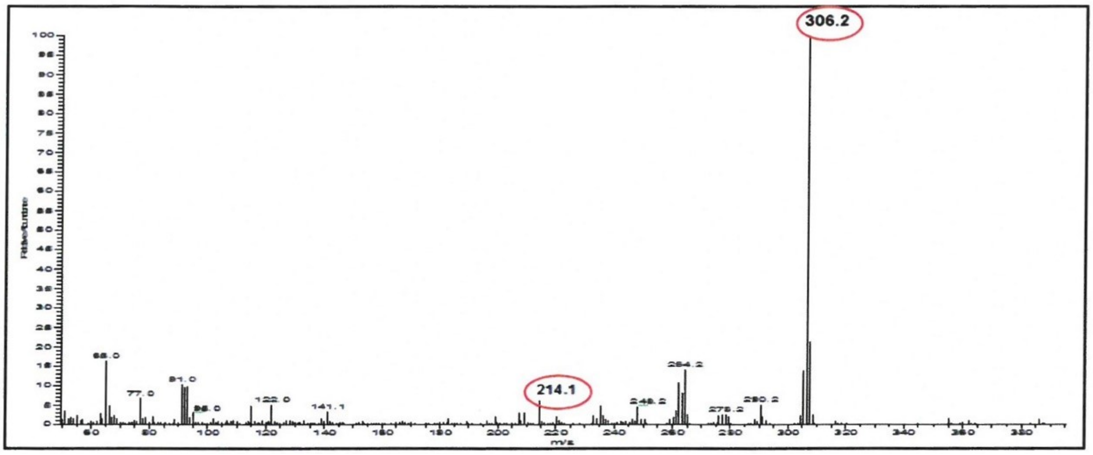

Figure S10. MS analysis of freshly prepared GP2.

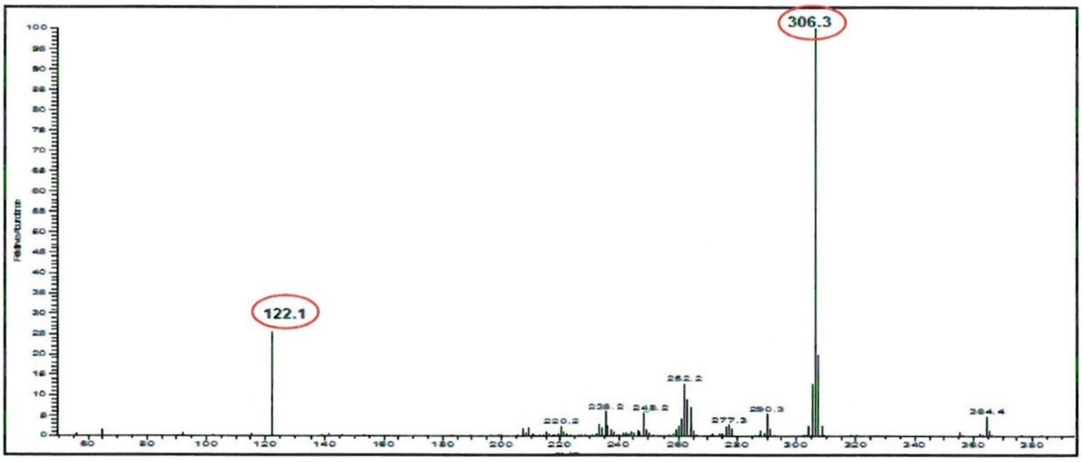

Figure S11. MS analysis of freshly prepared NP2.
